# Supplementary material for: Structural basis for Sarbecovirus ORF6 mediated blockage of nucleocytoplasmic transport
Source: Nat Commun. 2022 Aug 15;13:4782. doi: 10.1038/s41467-022-32489-5 (PMC9376891; doi:10.1038/s41467-022-32489-5)
Supplement: Supplementary file 1 — Supplementary Information [file 41467_2022_32489_MOESM1_ESM.pdf]

Supplementary Information for

Structural Basis for *Sarbecovirus* ORF6 Mediated Blockage of Nucleocytoplasmic Transport

Xiaopan Gao [1,#], Huabin Tian [2,#], Kaixiang Zhu [1,#], Qing Li [2,3], Wei Hao [1], Linyue Wang [1], Bo Qin [1], Hongyu Deng[2,3,*] and Sheng Cui [1,4*]

[1] NHC Key Laboratory of Systems Biology of Pathogens, Institute of Pathogen Biology, Chinese Academy of Medical Sciences and Peking Union Medical College, Beijing, 100730, P. R. China.

[2] CAS Key Laboratory of Infection and Immunity, CAS Center for Excellence in Biomacromolecules, Institute of Biophysics, Chinese Academy of Sciences, Beijing 100101, China.

[3] University of Chinese Academy of Sciences, Beijing 100049, China.

[4]Sanming Project of Medicine in Shenzhen, National Clinical Research Center for Infectious Diseases, Shenzhen Third People's Hospital, Southern University of Science and Technology

*Correspondence should be addressed to:

S.C. (cui.sheng@ipb.pumc.edu.cn) or H.D. (hydeng@moon.ibp.ac.cn.)

#These authors contributed equally to this work.

Supplementary Figures

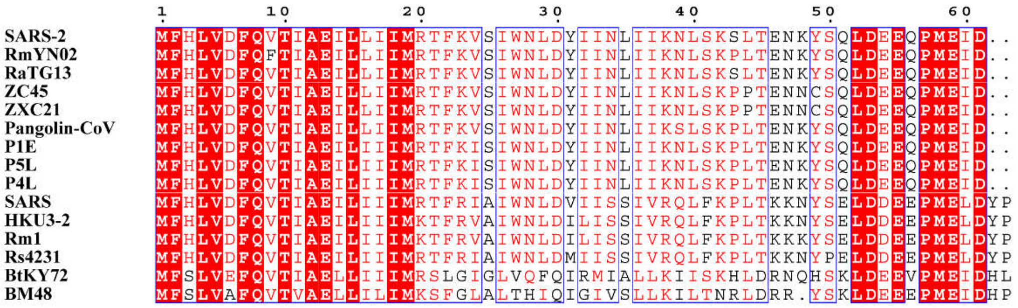

Supplementary Figure 1.Multiple sequence alignment of ORF6 from representative Sarbecovirus

SARS-CoV-2, SARS-CoV-2-related viruses from bats (RmYN02, RaTG13, ZXC21 and ZC45) and pangolins (pangolin-CoV,P1E,P5LP4L),SARS-CoV (Tor2), SARS-CoV-related viruses from bats (HKU3-2, Rm1 and Rs4231), the two bat sarbecoviruses that are phylogenetically located at the outgroup of SARS-CoV-2 and SARS-CoV (BtKY72 and BM48) are also included. The sequence alignment was calculated and rendered using the software Clustal Omega and ESPript 3.0.

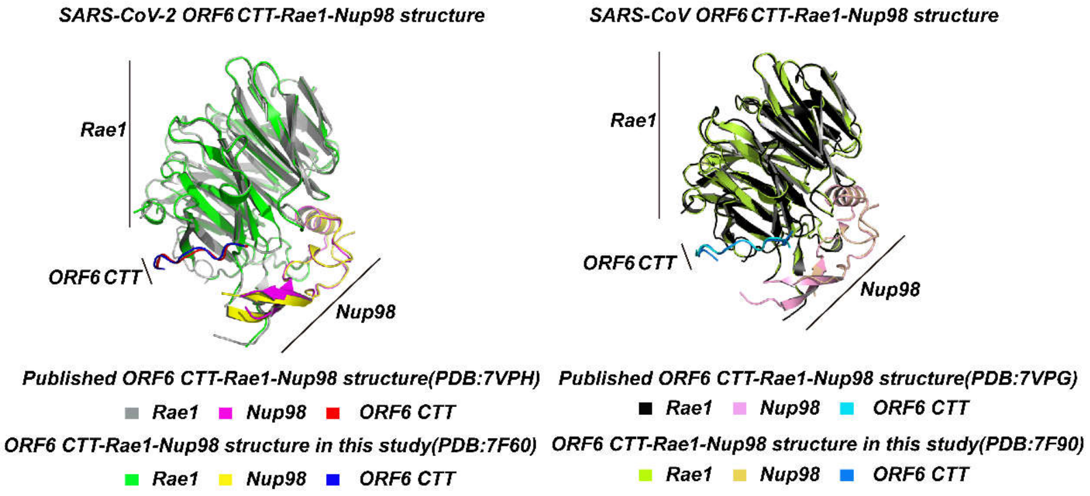

Supplementary Figure 2. Superimposition of the recently published ORF6 CTT-Rae1-Nup98 structure with the ORF6 CTT-Rae1-Nup98 structure in this study.

The published SARS-CoV-2 ORF6 CTT-Rae1-Nup98 structure (PDB ID: 7VPH) and SARS-CoV ORF6 CTT-Rae1-Nup98 structure (PDB ID: 7VPG) are superimposed with the SARS-CoV-2 ORF6 CTT-Rae1-Nup98 structure (PDB ID:7F60) and SARS-CoV ORF6 CTT-Rae1-Nup98 structure (PDB ID:7F90) in this study respectively.

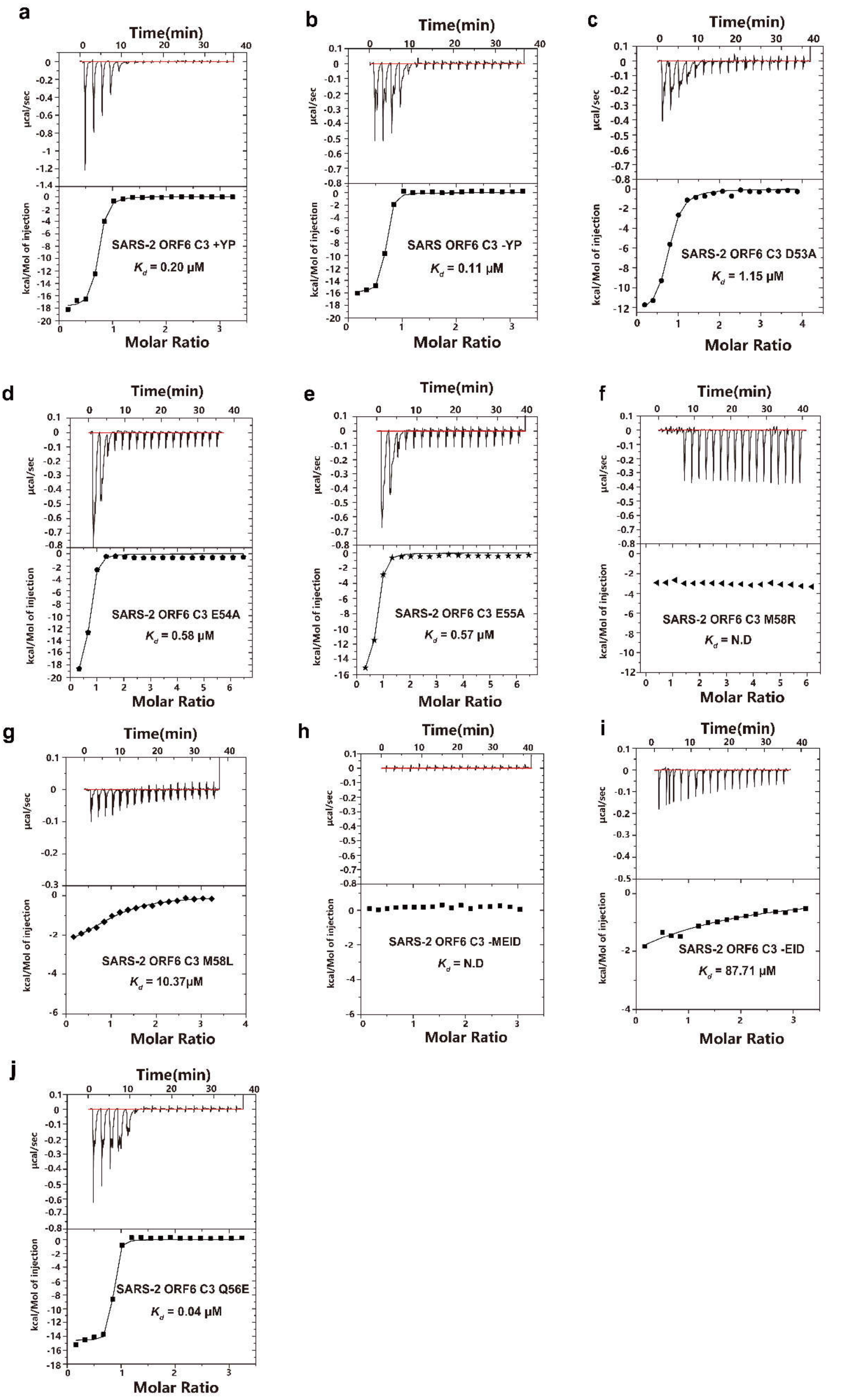

Supplementary Figure 3. ITC assay between ORF6 mutants peptide and Rae1-Nup98

complex

a-j. Binding isotherm for the interaction between ORF6 mutants peptide and Rae1-Nup98 complex. The calculated K_d values are indicated.

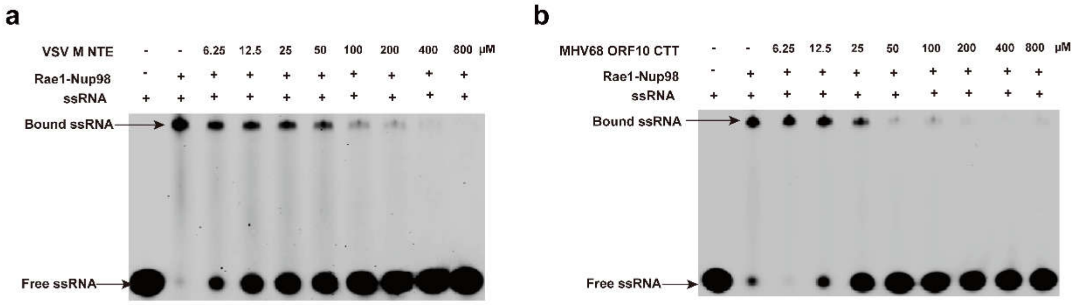

Supplementary Figure 4. EMSA experiments showing the ability of VSV M-NTE and MHV68 ORF10-CTT peptides dislocating ssRNA from Rae1-Nup98 complex

a-b. After pre-incubating FAM labeled 10-mer poly(U) ssRNA with Rae1-Nup98, increasing amount of peptides (VSV M NTE and MHV68 ORF10 CTT, indicted on top left of each gel) were added to the mixtures to allow RNA dislocation from Rae1-Npu98. The resulting mixtures were analyzed by Native-PAGE and visualized using Typhoon scanner. Results shown are representative of three independent experiments. Source data are provided as a Source data file.

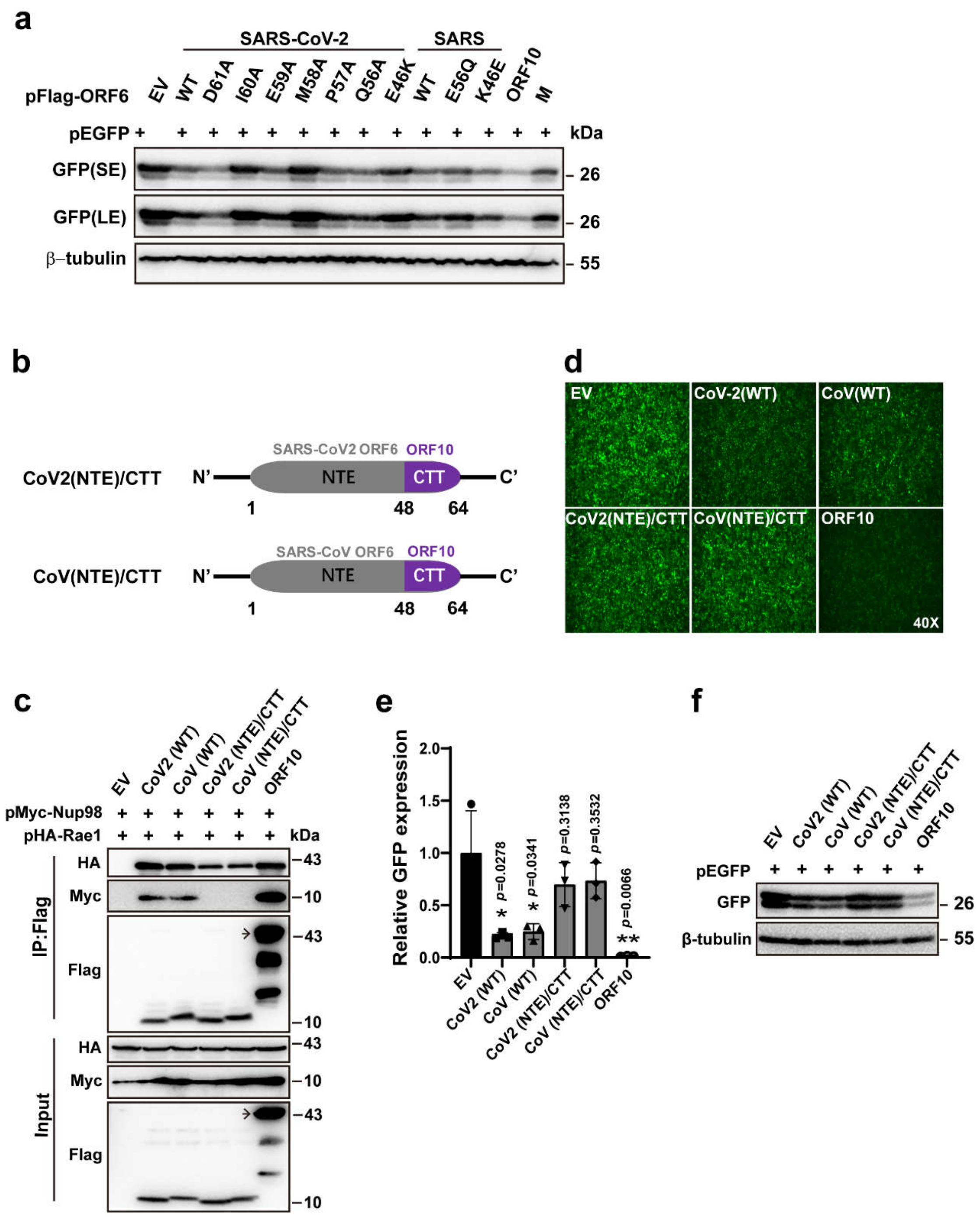

Supplementary Figure 5. The effect of ORF6 variants and hybrids on their interactions with Rae1-Nup98 and inhibition of GFP expression.

a) HEK293T cells were transfected with pEGFP-C1 and individual plasmid expressing ORF6 mutants, the effects of mutations on the GFP expression were analyzed by western blot. Results shown are representative of three independent experiments.

b) A schematic illustration of the chimeric ORF6 generated hybrids.

c) The interactions between ORF6 hybrids and Rae1-Nup98. HEK293T cells were transfected with the indicated plasmids expressing HA-tagged Rae1, Myc-tagged Nup98, and and Flag-tagged ORF6 hybrid or ORF10, and Co-IP was performed as described in the Methods. All data were based on at least three independent experiments

d) HEK293T cells were transfected with pEGFP-C1 and individual plasmid expressing ORF6 hybrids or ORF10, and the effects of ORF6 hybrids on GFP expression were analyzed by fluorescence microscopy. All fluorescence images at 40 times magnification (40 x). All data were based on at least three independent experiments.
e) Quantification of the fluorescence intensity in panel d with Image J. Date are representative of three independent experiments and shown as mean±SD. (* p<0.05, ** p<0.001; two tailed Student's t test, n=3).
f) The GFP expression in panel d were detected by western blotting. All data were based on at least three independent experiments. Source data are provided as a Source data file.

Supplementary Tables

Supplementary Table 1. Thermodynamic parameters of the binding between ORF6 synthetic peptides and Rae1-Nup98

Binding partners	*N*	*K_a, M^{-1}*	*K_d, μM*	*ΔH, cal/mole*	*ΔS, cal/mole/deg*
SARS-2 ORF6 C1 vs Rae1-Nup98	1.06	$2.62*10^6±5.73*10^5$	0.38	$-1.015*10^4±163.2$	-4.68
SARS-2 ORF6 C2 vs Rae1-Nup98	1.17	$3.58*10^6±4.27*10^5$	0.27	-9582±77.3	-2.13
SARS-2 ORF6 C3 vs Rae1-Nup98	0.9	$4.12*10^6±6.69*10^5$	0.24	$-1.221*10^4±140.8$	-10.7
SARS-2 ORF6 C4 vs Rae1-Nup98	0.82	$2.25*10^6±7.96*10^5$	0.44	$-1.250*10^4±404.6$	-12.8
SARS-2 ORF6 C3-D61A vs Rae1-Nup98	0.76	$3.61*10^5±8.07*10^4$	2.77	$-2.388*10^4±1277$	-54.6
SARS-2 ORF6 C3-I60A vs Rae1-Nup98	0.68	$2.38*10^5±4.00*10^4$	4.20	$-1.770*10^4±722.2$	-34.6
SARS-2 ORF6 C3-E59A vs Rae1-Nup98	0.68	$6.06*10^5±1.25*10^5$	1.65	$-1.639*10^4±664.8$	-28.3
SARS-2 ORF6 C3-M58A vs Rae1-Nup98	ND	ND	ND	ND	ND
SARS-2 ORF6 C3-M58R vs Rae1-Nup98	ND	ND	ND	ND	ND
SARS-2 ORF6 C3-M58L vs Rae1-Nup98	1.00	$9.64*10^4±1.31*10^4$	10.37	-2954±176.3	12.9
SARS-2 ORF6 C3-P57A vs Rae1-Nup98	2.34	$1.12*10^6±1.81*10^5$	0.89	-5479±74.9	9.30
SARS-2 ORF6 C3-Q56A vs Rae1-Nup98	0.96	$2.33*10^6±3.08*10^5$	0.42	$-1.132*10^4±132.7$	-8.83

SARS-2 ORF6 C3-Q56E vs Rae1-Nup98	0.781	$2.08*10^7±8.12*10^6$	0.04	$-1.460*10^4±176.1$	-15.5
SARS-2 ORF6 C3-E55A vs Rae1-Nup98	0.65	$1.75*10^6±4.24*10^5$	0.57	$-1.604*10^4±476.3$	-25.2
SARS-2 ORF6 C3-E54A vs Rae1-Nup98	0.59	$1.72*10^6±5.99*10^5$	0.58	$-1.994*10^4±821.4$	-38.3
SARS-2 ORF6 C3-D53A vs Rae1-Nup98	0.71	$8.63*10^5±1.26*10^5$	1.15	$-1.288*10^4±292.1$	-16.0
SARS-2 ORF6 C3-EID vs Rae1-Nup98	1.01	$1.14*10^4±7.68*10^3$	87.71	-7222±9347	-5.65
SARS-2 ORF6 C3-MEID vs Rae1-Nup98	ND	ND	ND	ND	ND
SARS-2 ORF6 C3+YP vs Rae1-Nup98	0.66	$4.93*10^6±6.15*10^5$	0.20	$-1.782*10^4±173.9$	-29.1
SARS ORF6 C3 vs Rae1-Nup98	0.63	$2.69*10^6±6.35*10^5$	0.37	$-1.279*10^4±293.8$	-13.5
SARS ORF6 C3-YP vs Rae1-Nup98	0.62	$8.89*10^6±1.78*10^6$	0.11	$-1.589*10^4±177.8$	-21.5
SARS ORF6 C3-E56Q vs Rae1-Nup98	1.30	$2.55*10^5±6.67*10^4$	3.92	$-1.122*10^4±712.4$	-12.9
MHV-68 ORF10-CTT vs Rae1-Nup98	1.37	$1.75*10^5±4.03*10^4$	5.71	-9760±547.9	-8.73
VSV M-NTT vs Rae1-Nup98	0.304	$8.55*10^3±2.76*10^3$	116.95	$-7.864*10^4±1.998*10^5$	-246

N, stoichiometry

Ka, affinity constant; standard deviation did not exceed ±10%.

Kd, dissociation constant; calculated as 1/Ka.

ΔH, enthalpy variation; standard deviation did not exceed ±10%.

ΔS, entropy variation.

ND, not determined

Supplementary Table 2

Data collection and refinement statistics

	SARS-CoV-2 ORF6 in complexed Rae1-Nup98 (PDB ID: 7F60)	SARS CoV ORF6 in complexed Rae1-Nup98 (PDB ID: 7F90)
**Data collection**		
Space group	C121	C121
Cell dimensions		
a, b, c (Å)	193.35, 87.53,48.23	189.33, 86.79,47.90
α, β, γ (°)	90.00,93.68, 90.00	90.00,92.08,90
X ray source		
Wavelength (Å)	0.978520	0.978520
Data range (Å)	48.27-2.85	47.87-2.39
Reflections unique	36291[a]	60024[a]
R_{sym} [b] (last shell)	0.372 (1.541)	0.131 (0.624)
I / σI (last shell)	3.47 (0.80)	7.62 (2.01)
Completeness (%) (last shell)	99.0 (97.4)	99.7 (99.2)
Redundancy (last shell)	3.53 (3.51)	3.49 (3.55)
**Refinement**		
Resolution range (Å)	48.24-2.85	47.87-2.39
Reflections, cut-off, % reflections in cross validation	18701[a], F>1.35, 4.83	30632[a], F>1.34, 5.22
R_{work} [c]/ R_{free} [d] (last shell)	0.2357/0.2756 (0.3425/0.3861)	0.2196/0.2581 (0.2838/0.3209)
**Atoms**		
Non-hydrogen protein atoms	6153	11937
Protein	6153	11937
Solvent	0	189
B-factors average (Å^2)	40.84	44.04
Protein (Å^2)	40.84	44.15
Ligands (Å^2)	0	0
Solvent (Å^2)	0	37.39
**r.m.s.d**		
Bond lengths (Å)	0.002	0.005
Bond angles (°)	0.487	0.737
% residues in favored regions, allowed regions, outliers in Ramachandran plot	97.36,2.64,0	95.54,4.46,0

Values in parentheses are for the highest-resolution shell.

[a]Friedel pairs are treated as different reflections

[b] $R_{sym} = \sum_{hkl}\sum_j |I_{hkl,j} - I_{hkl}|/\sum_{hkl}\sum_j I_{hkl,j}$, where I_{hkl} is the average of symmetry-related observations of a unique reflection

[c] $R_{work} = \sum_{hkl} ||F_{obs}(hkl)|-|F_{calc}(hkl)||/\sum_{hkl}|F_{obs}(hkl)|$.

[d] R_{free}= the cross-validation R factor for 5% of reflections against which the model was not refined.

Supplementary Table 3

Various ORF6 synthetic peptides used in this study

**Peptide #**	**Peptide name**	**Sequence (N' to C')**
**1**	SARS-CoV-2 ORF6 C1	**SLTENKYSQLDEEQPMEID**
**2**	SARS-CoV-2 ORF6 C2	**ENKYSQLDEEQPMEID**
**3**	**SARS-CoV-2 ORF6 C3**	**YSQLDEEQPMEID**
**4**	SARS-CoV-2 ORF6 C4	**DEEQPMEID**
**5**	SARS-CoV-2 ORF6-D61A	**YSQLDEEQPMEIA**
**6**	SARS-CoV-2 ORF6-I60A	**YSQLDEEQPMEAD**
**7**	SARS-CoV-2 ORF6-E59A	**YSQLDEEQPMAID**
**8**	SARS-CoV-2 ORF6-M58A	**YSQLDEEQPAEID**
**9**	SARS-CoV-2 ORF6-M58R	**YSQLDEEQPREID**
**10**	SARS-CoV-2 ORF6-M58L	**YSQLDEEQPLEID**
**11**	SARS-CoV-2 ORF6-P57A	**YSQLDEEQAMEID**
**12**	SARS-CoV-2 ORF6-Q56A	**YSQLDEEAPMEID**
**13**	SARS-CoV-2 ORF6-E55A	**YSQLDEAQPMEID**
**14**	SARS-CoV-2 ORF6-E54A	**YSQLDAEQPMEID**
**15**	SARS-CoV-2 ORF6-D53A	**YSQLAEEQPMEID**
**16**	SARS-CoV-2- ORF6-EID	**YSQLDEEQPM**
**17**	SARS-CoV-2- ORF6-MEID	**YSQLDEEQP**
**18**	SARS-CoV-2 ORF6+YP	**YSQLDEEQPMEIDYP**
**19**	**SARS-CoV ORF6 C3**	**YSELDDEEPMELDYP**
**20**	SARS-CoV ORF6-YP	**YSELDDEEPMELD**
**21**	SARS-CoV ORF6-E56Q	**YSELDDEQPMELDYP**
**22**	SARS-CoV-2 ORF6-Q56E	**YSQLDEEEPMEID**
**23**	MHV68-ORF10-CTT	**RAITDGGESPMEWQTL**
**24**	VSV-M-NTE	**DEMDTHDPNQLRY**

Supplementary Table 4

Optimized genes in this study

Gene names	Optimized genes sequence
Rae1	>ATGTCGCTATTCGGCACCACTAGCGGTTTTGGAACCTCTGGTACGTCCATG TTTGGGTCAGCGACCACTGATAACCACAATCCTATGAAGGACATCGAGGTC ACTAGCAGTCCGGATGACTCTATCGGCTGCCTTTCGTTCTCCCCGCCCACGC TGCCGGGAAACTTCCTAATCGCTGGTTCTTGGGCCAACGACGTGAGATGTT GGGAGGTTCAGGATAGTGGCCAGACGATCCCGAAGGCCCAGCAAATGCAT ACCGGGCCTGTTTTAGATGTGTGCTGGAGCGATGACGGGTCAAAAGTCTTT ACTGCGAGCTGTGATAAGACGGCGAAGATGTGGGACTTGTCGAGTAATCAG

	GCTATACAGATCGCTCAACATGATGCGCCCGTCAAGACAATACATTGGATAA<br>AGGCGCCCAACTACTCGTGTGTAATGACTGGTTCCTGGGACAAAACGCTCA<br>AATTCTGGGATACCCGTTCATCTAACCCCATGATGGTTCTTCAACTTCCAGA<br>ACGATGCTATTGTGCAGACGTCATATACCCGATGGCCGTAGTCGCTACAGCT<br>GAGCGAGGGCTCATCGTATACCAGCTGGAAAATCAACCATCAGAATTTAGG<br>CGTATAGAATCCCCTTTGAAGCACCAACACCGCTGTGTGGCCATTTTTAAAG<br>ACAAGCAAAATAAGCCCACTGGTTTTGCCCTCGGTTCTATTGAGGGCAGAG<br>TGGCCATACATTATATTAATCCTCCAAACCCTGCAAAAGACAATTTTACCTTC<br>AAATGCCATCGGTCTAATGGCACAAATACAAGTGCGCCACAAGATATATACG<br>CGGTAAACGGGATTGCTTTCCACCCAGTACACGGAACGCTGGCTACAGTTG<br>GTTCGGATGGCCGCTTTTCATTTTGGGACAAAGACGCAAGAACAAAGTTGA<br>AGACCAGCGAACAATTAGATCAGCCAATTAGCGCCTGCTGTTTCAACCACA<br>ACGGGAACATTTTTGCATATGCAAGTTCCTATGATTGGTCAAAGGGACATGA<br>GTTCTACAATCCCCAGAAAAAAAATTATATTTTCTTACGGAATGCAGCAGAG<br>GAATTAAAACCGAGGAACAAAAAATAA
Nup98	>CCAACCGGCACGACTATTAAGTTTAACCCCCCGACGGGTACTGACACAAT<br>GGTGAAAGCGGGGGTATCAACGAACATAAGTACAAAGCACCAATGCATCA<br>CCGCCATGAAAGAATACGAGAGCAAGTCCCTAGAAGAATTACGTCTGGAA<br>GATTATCAGGCAAATCGAAAATAA
VSV　M	>ATGAGCAGCCTGAAGAAGATCCTGGGGCTGAAGGGCAAGGGGAAGAAGT<br>CAAAGAAGCTGGGGATCGCCCCCCCCCCTACGAGGAGGATACAAACATG<br>GAGTACGCCCCCAGCGCCCCCATCGATAAGAGCTACTTCGGGGTGGATGAG<br>ATGGATACACACGATCCCAACCAGCTGAGGTACGAGAAGTTCTTCTTCACA<br>GTGAAGATGACCGTGAGGAGCAACAGGCCCTTCAGGACCTACAGCGACGT<br>GGCCGCCGCCGTGAGCCACTGGGACCACATGTACATCGGGATGGCCGGGA<br>AGAGGCCCTTCTACAAGATCCTGGCCTTCCTGGGGTCCAGCAACCTGAAGG<br>CCACCCCCGCCGTGCTGGCCGACCAGGGCCAGCCCGAATACCACGCCCAC<br>TGTGAGGGGAGGGCCTACCTGCCCCACCGGATGGGCAAGACACCCCCCAT<br>GCTGAACGTGCCAGAGCACTTCCGGAGGCCCTTCAACATCGGCCTGTACA<br>AGGGCACAATCGAGCTGACAATGACCATCTACGATGATGAGAGCCTGGAG<br>GCCGCCCCCATGATCTGGGATCACTTCAACAGCAGCAAGTTCAGCGACTTC<br>CGGGAGAAGGCCCTGATGTTCGGCCTGATCGTGGAGAAGAAGGCCAGCGG<br>CGCCTGGGTGCTGGATAGCGTGAGCCACTTCAAGTAA
MHV68 0RF10	>ATGGAGAAGAGCGCGCCGAGCATCATTCTGAACCACTGGTGCGTGACCTG<br>GCAGGGTCACCACTTCCTGTGCCGTAACCTGAGCAACATCAAAATTCTGAA<br>CCGTCGTAACGGCTATACCACCCTGGACCTGCCGCTGACCCTGGGTGATCT<br>GACCCAATATCGTCTGGCGCACGGCCTGAGCGAAAACCTGATGGCGCTGA<br>GCCCGTACAGCTGGACCATCCCGTTCCTGGTGAGCAGCAGCGAGACCCCG<br>GGTATCGAACTGCTGCCGAAGGTTATTAACGACTTTGGCACCCCGCTGAGC<br>CTGGCGATCAAAACCAACCTGCCGAGCATTCCGGCGCACCAGCTGCTGTTC<br>TACATCATTTTTCTGCGTCCGAGCCCGCTGACCAGCATGAGCTGCTATGCGC<br>GTCCGCTGAGCCTGGCGAGCACCCCGAGCACCAACGGTCTGTGCCAAAGC<br>GTGAGCGTTCTGGACAACAAGCCGGGCCTGCTGATTACCACCCCGCTGCAC<br>CGTGATCCGGCGAGCGGTAAATACACCAGCAACGTGCAGAGCCCGACCAC

	CTTCAACCTGTTTCGTGTTCTGTATATCAAGCTGAGCGGCGAGGAAGGCAT<br>GGTTAGCCAAAAGGTTAAACACCTGACCATTGACAAAGATAGCCTGCAGG<br>AAGGTTTCCTGCAACTGTGCCTGAACATGTGCGGCGTGAGCTATGAAACCC<br>TGCAGTGCGAGATCCTGCTGGAACTGGTTCAAGGTCCGACCAACTTCATTT<br>TTCCGGCGGCGTTCCCGCCGCCGGTGAGCCTGCCGCACCGTAACTGCATCG<br>AGCTGACCTGCGACACCGAACGTTGCCTGAAGCCGGGCGATGTTATGAAG<br>CTGAAACACCGTCTGCTGTACGAGCTGGGTCAGCGTGGCGATACCCCGCAA<br>AACGCGTTCCTGATCGTGGGTGCGCACAGCCCGGAAACCGTTTGGATCAGC<br>CCGAGCCTGTGGCTGCCGGGTCAGCCGCTGTATATCAACATCATTAACCTGA<br>GCCACAAGCCGCTGCTGCTGAGCCGTCACAGCATTCTGGCGCTGGCGATCC<br>CGATTAGCTATACCCGTGCGCCGGCGAGCGCGCATGAGGCGACCGACACCA<br>CCATCTGCTATAGCGGTAACAGCCGTGTGCTGACCTGCGGTGCGGCGCATG<br>TTCTGGAAGCGCACTTTAAACACCCGCCGATCACCAGCCGTGCGATTACCG<br>ATGGTGGCGAGAGCCCGATGGAATGGCAAACCCTGTAA
SARS-CoV-2 ORF6	>ATGTTTCATCTCGTTGACTTTCAGGTTACTATAGCAGAGATATTACTAATTAT<br>TATGAGGACTTTTAAAGTTTCCATTTGGAATCTTGATTACATCATAAACCTCA<br>TAATTAAAAATTTATCTAAGTCACTAACTGAGAATAAATATTCTCAATTAGAT<br>GAAGAGCAACCAATGGAGATTGATTAA
SARS-CoV ORF6	>ATGTTTCATCTTGTTGACTTCCAGGTTACAATAGCAGAGATATTGATTATCA<br>TTATGAGGACTTTCAGGATTGCTATTTGGAATCTTGACGTTATAATAAGTTCA<br>ATAGTGAGACAATTATTTAAGCCTCTAACTAAGAAGAATTATTCGGAGTTAG<br>ATGATGAAGAACCTATGGAGTTAGATTATCCATAA
CoV2(NTE)/CTT	>ATGTTTCATCTCGTTGACTTTCAGGTTACTATAGCAGAGATATTACTAATTAT<br>TATGAGGACTTTTAAAGTTTCCATTTGGAATCTTGATTACATCATAAACCTCA<br>TAATTAAAAATTTATCTAAGTCACTAACTGAGAATAAACGTGCGATTACCGA<br>TGGTGGCGAGAGCCCGATGGAATGGCAAACCCTGTAA
CoV(NTE)/CTT	>ATGTTTCATCTTGTTGACTTCCAGGTTACAATAGCAGAGATATTGATTATCA<br>TTATGAGGACTTTCAGGATTGCTATTTGGAATCTTGACGTTATAATAAGTTCA<br>ATAGTGAGACAATTATTTAAGCCTCTAACTAAGAAGAATCGTGCGATTACCG<br>ATGGTGGCGAGAGCCCGATGGAATGGCAAACCCTG TAA

Supplementary Table 5

List of primers used in this study

Name	Forward primer(5'-3')	Reverse primer(5'-3')
pFastbacDual-Rae1(31-368)	ATCACCCGGGATCTCGAGATG<br>GACATCGAGGTCACTAGCAG	CCCCCATCTCCCGGTACCTTATT<br>TTTTGTTCCTCGGTTTTAA
pFastbacDual-N-His-Nup(157-213)	ACCATCGGGCGCGGAATGTCG<br>TACTACCATCACCATCACCATC<br>ACGATTACGATATCCCAACGA<br>CCGAAAACCTGTATTTTCAGG<br>GCGGATCCCCAACCGGCACGA<br>CTATTAA	GTACTTCTCGACAAGCTTTTATT<br>TTCGATTTGCCTGATAATCTT
pFlag-SARS-CoV ORF6	CGGGATCCGCCACCATGTTTC	ATAAGAATGCGGCCGCTTAACC

	ATCTCGTGGACTT	GTCATGGTCTTTGTAGTCAGAGCCACCTCCGCC
pFlag-SARS-CoV-2 ORF6	CCAAGCTTGCCACCATGTTCCACCTGGTGGACTTTC	GCTCTAGATTAACCGTCATGGTCTTTGTAGTCAGATCCTCCTCCTCC
pFlag-M	CCAAGCTTGCCACCATGAGCAGCCTGAAG	GCTCTAGATCAACCGTCATGGTCTTTGTAGTCCTTGAAGTGGCTCACGCT
pFlag-SARS-CoV ORF6(E56Q)	GAGCTGGACGATGAGCAACCCATGGAACTCG	GCTCATCGTCCAGCTCGGAGTAGTTTTTCTT
pFlag-SARS-CoV ORF6(K46E)	TTCAAGCCCCTCACCGAGAAAAACTACT	CGGTGAGGGGCTTGAACAGCTGGCGGAC
pFlag-SARS-CoV-2 ORF6(D61A)	AGCCAATGGAGATCGCTGGAGGAGGAGGATCT	GCGATCTCCATTGGCTGCTCCTCGTCCA
pFlag-SARS-CoV-2 ORF6(I60A)	AGGAGCAGCCAATGGAGGCCGATGGAGGAGGAGG	GCCTCCATTGGCTGCTCCTCGTCCAGCTGAG
pFlag-SARS-CoV-2 ORF6(E59A)	AGGAGCAGCCAATGGCGATCGATGGAGG	GCCATTGGCTGCTCCTCGTCCAGCTGAG
pFlag-SARS-CoV-2 ORF6(M58A)	GACGAGGAGCAGCCAGCGGAGATCGATGG	GCTGGCTGCTCCTCGTCCAGCTGAGAATA
pFlag-SARS-CoV-2 ORF6(P57A)	CTGGACGAGGAGCAGGCAATGGAGATCG	CCTGCTCCTCGTCCAGCTGAGAATACTTATT
pFlag-SARS-CoV-2 ORF6(Q56A)	CAGCTGGACGAGGAGGCGCCAATGGAGAT	GCCTCCTCGTCCAGCTGAGAATACTTATT
pFlag-SARS-CoV-2 ORF6(E46A)	AGCAAGTCCCTGACCAAGAATAAGTATT	TGGTCAGGGACTTGCTCAGATTCTTGATG
