# Supplementary material for: Structural basis for Sarbecovirus ORF6 mediated blockage of nucleocytoplasmic transport
Source: Nat Commun. 2022 Aug 15;13:4782. doi: 10.1038/s41467-022-32489-5 (PMC9376891; doi:10.1038/s41467-022-32489-5)
Supplement: Supplementary file 3 — Reporting Summary [file 41467_2022_32489_MOESM3_ESM.pdf]

nature portfolio

Corresponding author(s): Sheng CUI

Last updated by author(s): Jul 20, 2022

Reporting Summary

Nature Portfolio wishes to improve the reproducibility of the work that we publish. This form provides structure for consistency and transparency in reporting. For further information on Nature Portfolio policies, see our Editorial Policies and the Editorial Policy Checklist.

Statistics

For all statistical analyses, confirm that the following items are present in the figure legend, table legend, main text, or Methods section.

n/a	Confirmed	
☐	☒	The exact sample size (*n*) for each experimental group/condition, given as a discrete number and unit of measurement
☐	☒	A statement on whether measurements were taken from distinct samples or whether the same sample was measured repeatedly
☐	☒	The statistical test(s) used AND whether they are one- or two-sided<br>*Only common tests should be described solely by name; describe more complex techniques in the Methods section.*
☒	☐	A description of all covariates tested
☒	☐	A description of any assumptions or corrections, such as tests of normality and adjustment for multiple comparisons
☐	☒	A full description of the statistical parameters including central tendency (e.g. means) or other basic estimates (e.g. regression coefficient) AND variation (e.g. standard deviation) or associated estimates of uncertainty (e.g. confidence intervals)
☐	☒	For null hypothesis testing, the test statistic (e.g. *F*, *t*, *r*) with confidence intervals, effect sizes, degrees of freedom and *P* value noted<br>*Give P values as exact values whenever suitable.*
☒	☐	For Bayesian analysis, information on the choice of priors and Markov chain Monte Carlo settings
☒	☐	For hierarchical and complex designs, identification of the appropriate level for tests and full reporting of outcomes
☒	☐	Estimates of effect sizes (e.g. Cohen's *d*, Pearson's *r*), indicating how they were calculated

Our web collection on statistics for biologists contains articles on many of the points above.

Software and code

Policy information about availability of computer code

Data collection	BL19U1 beamline of National Facility for Protein Science in Shanghai (NFPS), the staff of the BL17U1 beamline and the staff of the BL10U2 beamline at the Shanghai Synchrotron Radiation Facility for assistance in data collection.<br>The isothermal titration calorimetry(ITC) measurement was performed using MicroCal iTC200 calorimeter (MicroCal, USA)<br>Typhoon Trio Variable Mode Imager (GE healthcare)was used to visualize the fluorescence signal in EMSA assay.<br>Fluorescence images were obtained using A1R+ (Nikon) and Olympus CKX41.<br>All western blot images were scanned and collected using MINICHEMI (SAGECREATION, China).<br>RT-PCR data were acquired with Quantstudio 7 (Applied Biosystems).<br>The luciferase activity was detected with GloMax-Multi JR detection system (Promega).
Data analysis	Origin Version 9 ;XDS package Version20190315,Coot version 0.8.9.2,CCP4 version 7.1(Including Phaser MR Version 2.5.6) and Phenix version 1.19.2-4158 were used for structure determination. For analysis of crystal structure,Pymol version 2.4.1 was used. NIS-Elements viewer 4.20 was used for confocal images analysis. Image J version 1.8.0 for western blot and GFP fluorescence intensity analysis. Graphpad Prism (version 8) was used for data analysis.

For manuscripts utilizing custom algorithms or software that are central to the research but not yet described in published literature, software must be made available to editors and reviewers. We strongly encourage code deposition in a community repository (e.g. GitHub). See the Nature Portfolio guidelines for submitting code & software for further information.

Data

Policy information about availability of data

All manuscripts must include a data availability statement. This statement should provide the following information, where applicable:

- Accession codes, unique identifiers, or web links for publicly available datasets
- A description of any restrictions on data availability
- For clinical datasets or third party data, please ensure that the statement adheres to our policy

The atomic coordinates and structure factors have been deposited in the Protein Data Bank under the accession codes: 7F60 (SARS-CoV-2 ORF6 in complexed Rae1-Nup98) [https://doi.org/10.2210/pdb7F60/pdb] and 7F90 (SARS CoV ORF6 in complexed Rae1-Nup98) [https://doi.org/10.2210/pdb7F90/pdb]. Publicly available protein atomic models with the following PDB code was used in the study:4OWR. Other data are available from the corresponding author upon reasonable request. Source data are provided with this paper.

Field-specific reporting

Please select the one below that is the best fit for your research. If you are not sure, read the appropriate sections before making your selection.

[x] Life sciences   [ ] Behavioural & social sciences   [ ] Ecological, evolutionary & environmental sciences

For a reference copy of the document with all sections, see nature.com/documents/nr-reporting-summary-flat.pdf

Life sciences study design

All studies must disclose on these points even when the disclosure is negative.

Sample size	Prior sample size determination was not done. No statistical methods were used to predetermine sample size. Sample size was determined according to our experience as well as literature reporting in terms of specific experiment. For cell studies ,the control samples or experimental samples both consisting of three independent samples. For EMASA and ITC assay, The experiment was performed in three independent experiments, with similar results. For statistical analysis, all experiments were performed in biologically independent triplicates to ensure robustness of the analysis and conclusions. Where statistical tests were applied, we selected n=3 as the minimum sample size. These sample sizes are seen as sufficient since no inference from small sample to larger populations is made.
Data exclusions	No data was excluded during analysis.
Replication	All the presented experimental results were performed at least three times independently. All attempts at replication were successful. Immunofluorescence assay was performed in biological independent duplicates with similar results.
Randomization	No randomization was performed because it was not necessary in our experimental design, and there are no groups to be allocated. In addition, the experimental results were not randomize. For cell experiments, cells from the mother flask were randomly placed in different cell dishes, each experiment were representative of three independent experiments. Because all groups were derived from the same cell line, no randomization could be performed.
Blinding	Blinding is not relevant to this study,we allocated samples into pre-determined experimental groups with the intention of studying the differences between them. only one variable is tested in each experiment. However,the investigators were blinded during the sample collection and data analysis (crystal data collection and structure determination,EMSA assay,ITC assay,Immunofluorescence, western blot, RT-PCR and luciferase detection).

Reporting for specific materials, systems and methods

We require information from authors about some types of materials, experimental systems and methods used in many studies. Here, indicate whether each material, system or method listed is relevant to your study. If you are not sure if a list item applies to your research, read the appropriate section before selecting a response.

Materials & experimental systems

n/a	Involved in the study	
[ ]	[x]	Antibodies
[ ]	[x]	Eukaryotic cell lines
[x]	[ ]	Palaeontology and archaeology
[x]	[ ]	Animals and other organisms
[x]	[ ]	Human research participants
[x]	[ ]	Clinical data
[x]	[ ]	Dual use research of concern

Methods

n/a	Involved in the study	
[x]	[ ]	ChIP-seq
[x]	[ ]	Flow cytometry
[x]	[ ]	MRI-based neuroimaging

Antibodies

Antibodies used	Anti-Flag antibody (Sigma-Aldrich, Cat#:F7425, 1:1000), anti-HA antibody (Sigma-Aldrich, Cat#:H6908, 1:1000), anti-Myc antibody (Sigma-Aldrich, Cat#:SAB4300319, 1:1000), Anti-FLAG M2 affinity Gel (Merck, Cat#:A2220), anti-Lamin A/C (Cell Signaling technology, Cat#:4777S, 1:2000), anti-STAT1 (Cell Signaling technology, Cat#:14994, 1:1000), anti-p-STAT1 (Cell Signaling technology, Cat#:9177S, 1:100), anti-GAPDH (Huaxing bio, Cat#:HX1828, 1:2000), Goat anti-Mouse IgG (H+L) Highly Cross-Adsorbed Secondary Antibody, Alexa Fluor 488 (Invitrogen, Cat#:A11029, 1:500), Goat anti-Rabbit IgG (H+L) Cross-Adsorbed Secondary Antibody, Alexa Fluor 555 (Invitrogen, Cat#:A21428, 1:500).
Validation	All antibodies were purchased from commercial companies and have been validated by the companies.Antibodies were further validated by using positive and negative controls in our studies, or based on correct molecular masses. Please see company's websites for the details.<br>Anti-Flag antibody (Sigma-Aldrich, Cat#: F7425, 1:1000): https://www.sigmaaldrich.cn/CN/zh/product/sigma/f7425<br>anti-HA antibody (Sigma-Aldrich, Cat#: H6908, 1:1000): https://www.sigmaaldrich.cn/CN/zh/product/sigma/h6908<br>anti-Myc antibody (Sigma-Aldrich, Cat#: SAB4300319, 1:1000): https://www.sigmaaldrich.cn/CN/zh/product/sigma/sab4300319<br>Anti-FLAG M2 affinity Gel (Merck, Cat#: A2220): https://www.sigmaaldrich.cn/CN/zh/product/sigma/a2220<br>anti-Lamin A/C (Cell Signaling technology, Cat#: 4777S, 1:2000): https://www.cellsignal.cn/products/primary-antibodies/lamin-a-c-4c11-mouse-mab/4777?site-search-type<br>anti-STAT1 (Cell Signaling technology, Cat#: 14994S, 1:1000): https://www.cellsignal.cn/products/primary-antibodies/stat1-d1k9y-rabbit-mab/14994?site-search-type<br>anti-p-STAT1 (Cell Signaling technology, Cat#: 9177S, 1:100): https://www.cellsignal.cn/products/primary-antibodies/phospho-stat1-ser727-antibody/9177?site-search<br>anti-GAPDH (Huaxing bio, Cat#: HX1828, 1:2000): http://www.huaxingbio.com/pd.jsp?id=52&nSL=%5B5%2C6%2C7%5D#skeyword=gapdh&_pp=0_35<br>Goat anti-Mouse IgG (H+L) Highly Cross-Adsorbed Secondary Antibody, Alexa Fluor 488 (Invitrogen, Cat#: A11029, 1:500): https://www.thermofisher.cn/cn/zh/antibody/product/Goat-anti-Mouse-IgG-H-L-Highly-Cross-Adsorbed-Secondary-Antibody-Polyclonal/A-11029<br>Goat anti-Rabbit IgG (H+L) Cross-Adsorbed Secondary Antibody, Alexa Fluor 555 (Invitrogen, Cat#: A21428, 1:500): https://www.thermofisher.cn/cn/zh/antibody/product/Goat-anti-Rabbit-IgG-H-L-Cross-Adsorbed-Secondary- Antibody-Polyclonal/A-21248.

Eukaryotic cell lines

Policy information about cell lines

Cell line source(s)	HEK-293T and HEK-293 cells(ATCC)
Authentication	HEK-293T and HEK293 cells were authenticated by ATCC. We will discard the cells after passing 15-20 passages, and recover new cells from frozen stocks. Cell morphology was monitored at each passage by microscope.
Mycoplasma contamination	All cell lines were mycoplasma-free. All cell lines are routinely tested for mycoplasma contamination every three months.
Commonly misidentified lines (See ICLAC register)	No commonly misidentified lines were used in this study.
